# Supplementary material for: ANT-Mediated Inhibition of the Permeability Transition Pore Alleviates Palmitate-Induced Mitochondrial Dysfunction and Lipotoxicity
Source: Biomolecules. 2024 Sep 15;14(9):1159. doi: 10.3390/biom14091159 (PMC11430505; doi:10.3390/biom14091159)
Supplement: Supplementary file 1 [file biomolecules-14-01159-s001.zip › biomolecules-3195596-supplementary.pdf]

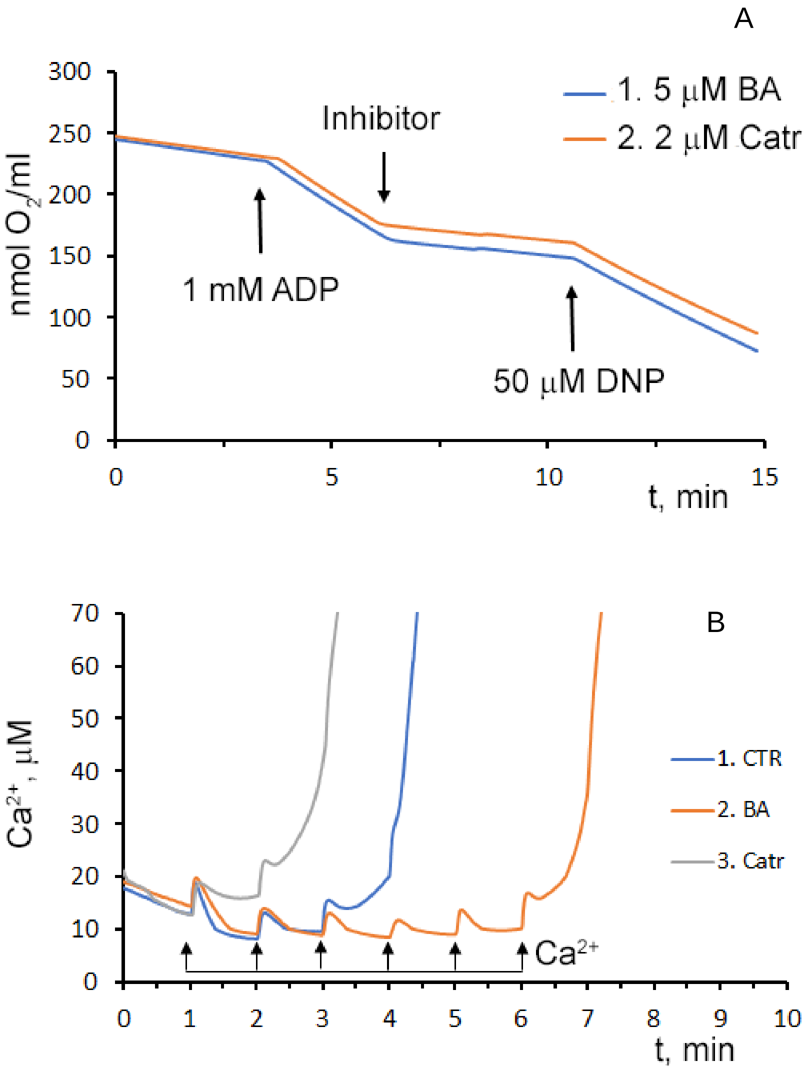

Figure S1: A) Representative recordings of changes in the respiration rate of rat liver mitochondria induced by bongkrekic acid (1) and carboxyatractyloside (2). 2.5 mM potassium glutamate + 2.5 mM malate was used as a respiration substrate. Mitochondrial protein concentration – 0.5 mg/ml. Additions: 1 mM ADP, 50 μM DNP. Typical traces are shown (*n* = 5). B) Calcium retention capacity of rat liver mitochondria in the presence of 5 μM bongkrekic acid (2) and 5 μM carboxyatractyloside (3). The figure shows the effect of successive additions of 10 μM $CaCl_2$ pulses. 2.5 mM potassium glutamate and 2.5 mM potassium malate were used as a respiratory substrate. Mitochondrial protein concentration – 0.5 mg/ml. Typical traces are shown (*n* = 4).

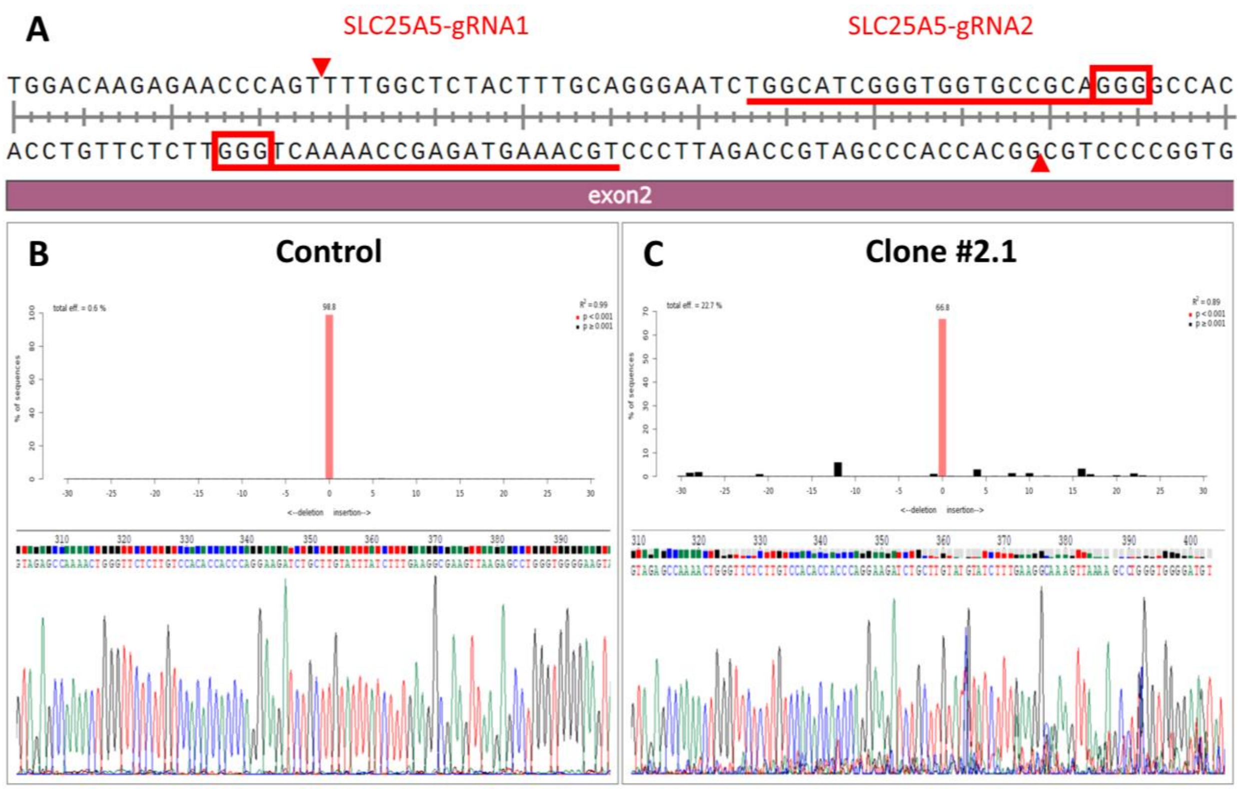

Figure S2. Design of gRNA protospacers to the SLC25A5 gene (A) and results of analysis of its nucleotide sequence in Control/untreated (B) and SLC25A5-gene edited (C) HEK293T cell clones. In panel A: gRNA protospacers are underlined with the red line, the corresponding PAM sequences are marked with red boxes and the cut sites are marked with red wedges. In panels B and C: top - the results of analysis with "TIDE: Tracking of Indels by DEcomposition" software [doi: 10.1093/nar/gku936]; bottom - the summation of the edited SLC25A5 alleles visualized with Chromas 2.6.6 software.

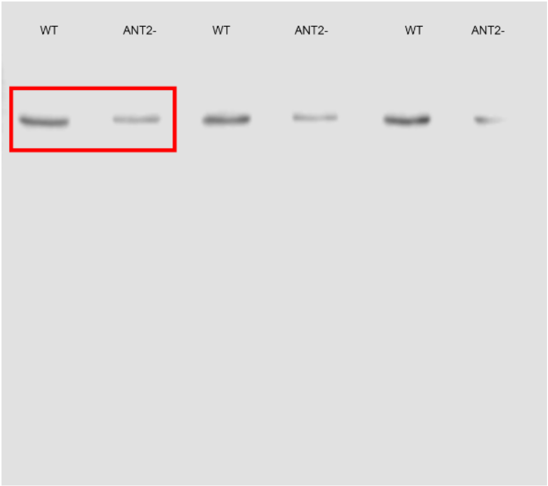

A

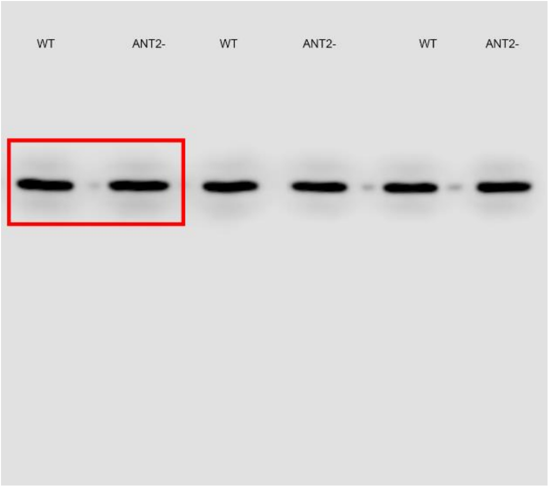

B

Figure S3. Full unedited WB membranes for Figure 6A. Western blotting of ANT2 (Abclonal A15639) (A) and GAPDH (Abclonal AC001) (B) proteins of HEK293T cells with normal (WT) and reduced expression (ANT2-, clone 2.1) of ANT2. The red rectangle marks the area used in Fig. 6A.
